# Alternative Therapies for the Prevention of Postoperative Nausea and Vomiting

**DOI:** 10.3389/fmed.2015.00087

**Published:** 2015-12-16

**Authors:** Nicoleta Stoicea, Tong J. Gan, Nicholas Joseph, Alberto Uribe, Jyoti Pandya, Rohan Dalal, Sergio D. Bergese

**Affiliations:** ^1^Department of Anesthesiology, The Ohio State University Wexner Medical Center, Columbus, OH, USA; ^2^Department of Anesthesiology, Stony Brook University Medical Center, Stony Brook, NY, USA; ^3^Department of Neuroscience, The Ohio State University, Columbus, OH, USA; ^4^Department of Psychology, The Ohio State University, Columbus, OH, USA; ^5^Department of Neurological Surgery, The Ohio State University Wexner Medical Center, Columbus, OH, USA

**Keywords:** postoperative nausea and vomiting, acupuncture, acupressure, hypnosis, alternative medicine

## Abstract

Postoperative nausea and vomiting (PONV) is a complication affecting between 20 and 40% of all surgery patients, with high-risk patients experiencing rates of up to 80%. Recent studies and publications have shed light on the uses of alternative treatment for PONV through their modulation of endogenous opioid neuropeptides and neurokinin ligands. In addition to reducing PONV, hypnosis was reported to be useful in attenuating postoperative pain and anxiety, and contributing to hemodynamic stability. Music therapy has been utilized to deepen the sedation level and decrease patient anxiety, antiemetic and analgesic requirements, hospital length of stay, and fatigue. Isopropyl alcohol and peppermint oil aromatherapy have both been used to reduce postoperative nausea. With correct training in traditional Chinese healing techniques, acupuncture (APu) at the P6 acupoint has also been shown to be useful in preventing early PONV, postdischarge nausea and vomiting, and alleviating of pain. Electro-acupuncture (EAPu), as with APu, provided analgesic and antiemetic effects through release and modulation of opioid neuropeptides. These non-pharmacological modalities of treatment contribute to an overall patient wellbeing, assisting in physical and emotional healing.

## Postoperative Nausea and Vomiting

Postoperative nausea and vomiting (PONV) is a complication affecting between 20 and 40% of all surgery patients, with high-risk patients experiencing rates of up to 80% (1–3). This condition severely affects patient satisfaction, with postoperative vomiting (POV) ranking above postoperative pain in least-desirable outcome. Moreover, while postoperative nausea (PON) is responsible for increasing patient anxiety, POV can cause more serious complications, including hematoma, dehydration, aspiration, and esophageal damage (1). For maxillofacial surgical patients, POV is responsible for wound dehiscence and bleeding, leading to further complications (1). In addition to these PONV-precipitated problems, emetic episodes can delay patient discharge from the post-anesthesia care unit by at least 20 min, with a residual group of patients experiencing severe PONV and requiring unplanned hospitalization (1, 3, 4).

## Neurophysiology

Stimulation of vestibular nuclei (VNU), area postrema (AP), and vagal afferent fibers from the gastrointestinal tract, project to the nucleus of the solitary tract (NST), with output pathways to local brain stem areas responsible for the vomiting reflex. NST sends projections to the mid- and fore-brain center responsible for the perception of nausea. Importantly, the AP’s capillaries are not surrounded by astrocytes and other glial cells comprising the blood–brain barrier (BBB), thus allowing input from toxins, metabolites, and drugs such as opioids, circulating within the systemic blood flow. The AP can then release serotonin, dopamine, acetylcholine, histamine, and neurokinin-1 (“substance-P”) to the NST as well as the reticular formation. Anesthesia is thought to modulate PONV by binding to the ionotropic 5-hydroxytryptamine type 3 (5-HT_3_) receptors within the AP and VAF, in addition to binding the solitary tract nucleus directly. Perioperative opioid administration has the potential to produce nausea and vomiting through binding to the μ-opioid receptor within the AP and NTS (5, 6).

## Risk Factors

Several risk factors exist for both PON and POV. Among them are: female gender, type of surgery, use of nitrous oxide, history of PONV or motion sickness, and perioperative opioid use. Female gender is widely considered to be the greatest risk factor for PON, but not for POV, with females experiencing three to four times the PON rate of male counterparts (1–3). Abdominal and ear, nose, and throat procedures are considered “high-risk” for PONV along with breast surgery, though the latter is believed to be due in part to the female gender. Interestingly, smoking status has been shown to decrease PONV in a manner dependent upon the time since the last cigarette. Whether the protective nature of smoking against PONV is due to nicotine in the tobacco or other chemical content has yet to be established. PONV also seems to be more prevalent in younger patients, especially those whose siblings or parents have experienced PONV (1–3). Iqbal et al. found that PONV steadily rose in prevalence for age 0–3, and thereafter decreased until age 14 when the prevalence again peaked. The authors then noted a steady decrease in PONV risk by around 10% per decade of life (3).

Postoperative opioid consumption also has a significant effect on the rate of PONV, Roberts et al. finding a consistent 6.0% POV reduction when postoperative opioid dose was reduced by half. The study also concluded that postoperative opioid dose escalation increased POV logarithmically (*r*^2^ = 0.98, *P* < 0.01) (7). Per Apfel et al., postoperative opioid use might be the single strongest predictor of PONV, with no difference between morphine and piritramide use postoperatively (8). Pierre et al. found patients with a previous history of PONV or motion sickness were four times more likely to experience PONV again, if opioids were required after the procedure (9). A consensus guideline developed by Gan et al. also found postoperative opioid use to be one of the most likely independent predictors of PONV. The guideline also suggested the contribution of alternative medicine to PONV and pain management (10).

## Antiemetics

Antiemetics have widely been used to manage PONV and are commonly given both as treatment and prophylaxis for at-risk patients. Several anti-emetic pharmacological classes are available including histamine antagonists, muscarinic acetylcholine antagonists, dopamine antagonists, corticosteroids, 5-HT_3_ antagonists, and tachykinin 1 antagonists. 5-HT_3_ receptor antagonists are the most commonly used type of antiemetic, with ondansetron being the most prescribed type. Droperidol, a selective dopamine receptor antagonist, dexamethasone, a corticosteroid, transdermal scopolamine, a non-selective, muscarinic acetylcholine receptor antagonist, and the newer 5-HT_3_ antagonist, palonosetron, have all been used to treat PONV (5).

## Alternative Therapies

Since 1784, when a commission from The Royal Academy of Science concluded that “imagination” was behind the treatment introduced by Franz Anton Mesmer to heal patients, alternative medicine has evolved rapidly, alongside mainstream medicine (11). For the past two centuries, hypnosis, relaxation imagery, music therapy, aromatherapy, acupressure (Apr), acupuncture (APu), and electroacupuncture (EAPu) have been studied and used mainly for anesthesia and analgesia in medical and surgical procedures. Recent studies and publications have shed light on the uses of alternative treatment for PONV, through their modulation of endogenous opioid neuropeptides and neurokinin ligands (12, 13).

### Hypnosis Therapy

At the end of the nineteenth century, Charcot demonstrated hypnosis on hysterical patients. His work was refuted by many contemporary scientists but recognized by renown psychiatrists like Sigmund Freud (1885) and Pierre Janet (14). Differing from its popular media portrayal, hypnosis has been used in the perioperative clinical settings to alleviate both postoperative pain and PONV (15). During hypnosis therapy, the caregiver, trained in hypnotic techniques, offers concentration strategies to the patient during the *induction* phase. These strategies allow the patient to more easily enter subsequent states of narrowed awareness. During the next stage of hypnosis, referred to as *deepening*, the hypnotist attempts to deepen the trance with imagery and memory recall with a focus on relaxation. During this trance state, known as *dissociation*, the patient experiences a separation of behavioral components, when, in a dream-like state, he or she becomes an observer while re-experiencing autobiographical memories. In this state, the hypnotist makes suggestions to reinforce feelings of relaxation and well-being, incompatible with pain or vomiting (12, 16). In a pediatric case report published in 2007, Mackenzie and Frawley described a four session hypnotherapy process used to treat PONV in a 6-year-old boy undergoing multiple esophageal dilation interventions. During these sessions, the patient received the suggestion that he had a “button in his brain” able to turn chewing, gagging, or nausea, ON or OFF. Nurses were instructed not to act in anticipation of a retching event (holding a kidney dish). The report noted a significant reduction and eventual cessation of PONV symptoms in this child (16). In a larger, randomized study of 100 thyroidectomy patients, Eberhart et al. played a tape recording of therapeutic suggestions intraoperatively. The study found a PONV rate reduction from 85.7% in the control group, to 47.2% in the treatment group. A substantial reduction in antimetic pharmacological requirements was reported as a result of this intervention (30.6 versus 68.6%). Studies in other populations have similarly found positive results with hypnosis for PONV (15). In addition to reducing PONV, hypnosis was reported to be useful in attenuating postoperative pain, anxiety, and contributing to hemodynamic stability (12). While the exact neuro-pathway by which hypnosis provides relief of PONV has yet to be fully elucidated, the area of the brain activated in a hypnotic state are known through positron emission tomography (PET) and magnetic resonance imaging studies. Utilizing PET imaging, Faymonville et al. found acute changes in both temporal lobes, basal forebrain areas, the parietal lobe, the precentral gyrus, the prefrontal cortex, insular, and cingulate cortices. Importantly, the medial parietal cortex demonstrated greatly reduced activity, similar to the decreases seen during unconsciousness, coma, general anesthesia, and rapid eye and non-rapid eye movement sleep. The anterior cingulate cortex (Brodmann area 24) was strongly activated during hypnotic trance. This area is known to be innervated by a wide variety of opioid, dopaminergic, seretonergic, noradrenergic, neurokinin 1, and other peptidic pathways. Hypnotic activation of the insulae and anterior cingulate cortex has been consistently shown during pain states. Crosstalk between these two structures and the midbrain and thalamus may provide descending pain inhibition through modification of the pain threshold and perception of pain intensity. The clinically observed analgesic effects of hypnosis may be thus explained through these pathways (12).

### Guided Imagery and Relaxation Therapy

Guided imagery is a highly personalized form of therapy wherein patients create a relaxing imagery scenario to relieve anxiety and pain. The most frequently encountered scenarios were linked to nature, a trip, family, the patient’s home, or a personal hobby or skill. Guided muscle relaxation may also be used prior to the patient envisioning his or her scenario to further relaxation. A recent meta-analysis performed by Sing and Dalmar in 2014 found that guided imagery intervention is effective for reducing anxiety and pain in surgical patients (17). A randomized study of elective colorectal surgery patients reported a perioperative reduction of opioid consumption by 50%. Such therapy is cost-effective and may be instituted as a complementary method of analgesic and anxiolytic care (18).

### Music Therapy

In addition to hypnotic therapy, music therapy has been utilized to deepen the sedation level and decrease patient anxiety, antiemetic and analgesic requirements, hospital length of stay, and fatigue (18, 19). A randomized, controlled study of 35 patients undergoing surgeries with spinal anesthesia and patient-controlled intravenous propofol sedation found a significant reduction in propofol use for the group exposed to music during surgery compared to the control group (18). Other studies found no benefit to music therapy, or reduced anxiolytic effect as compared with drug therapy. Thus, this form of therapy’s effectiveness is a subject of contention within the literature. Kliempt et al. utilized both hypnotic suggestion and synchronized hemispheric sound during surgery, as did Nilsson et al. (19). The therapy utilized two sounds, one per ear, with a difference in frequency of 1–30 Hz. The beats are generally imperceivable by the patient but may aid in synchronizing activity between the two hemispheres of the brain (20). Both studies noted a statistically significant reduction in analgesic usage. Nilsson also reported a decrease in the number of patients fatigued at discharge (19). However, no differences in the PONV rates between groups (interventional versus control) were noted. The exact mechanism by which music exerts its positive effects has yet to be conclusively determined. Lin linked heightened levels of growth hormone, and decreased levels of epinephrine and interleukin-6, with lower heart rates, blood pressure, and stress levels in patients exposed to music. Music may produce these changes through immune system mediator interactions with the pituitary gland, hypothalamus, and medulla (18, 21).

### Aromatherapy

Another form of adjuvant PONV therapy is aromatherapy, adopted from the traditional use of herbal medicine to treat a wide variety of ailments. Isopropyl alcohol and peppermint oil have both been used previously in studies to reduce PONV. The oil or alcohol is administered with a cotton swab placed under the nares and costs around $0.01 dollar per swab, one of aromatherapy’s benefits. Another benefit is a rapid patient response, which has been shown to be faster than the response to 4 mg ondansetron (21). Success of aromatherapy in treating nausea has differed amongst studies, even in similar populations. Merritt et al. found no difference in the rate of PONV with aromatherapy in ambulatory surgery patients, while Anderson and Gross found significant reduction in a similar surgical populations (21, 22). Of interest, Anderson and Gross found no difference between the interventional groups receiving alcohol, peppermint oil, and the placebo saline group. The authors suggested that patient anticipation of PONV symptom alleviation may be responsible for the positive effects of aromatherapy more so than any pharmacodynamic effect elicited from oil or alcohol use (22). Tate noted significant benefit to aromatherapy in gynecological patients, where Ferruggiari found no significant benefit in a general population of female surgical patients (21, 23). Wang et al. found limited efficacy of aromatherapy when treating PONV in pediatric patients. Further studies are necessary to better elucidate the benefits of aromatherapy; however, the American Society of PeriAnesthesia Nurses (ASPAN) *2006 Evidence-Based Clinical Practice Guidelines for the Prevention and/or Management of PONV* noted that the benefits of such therapy equal the risks. A 2012 Cochrane Library review also recognized aromatherapy as generally effective (21, 24).

### Acupressure

Acupressure is believed to function by increasing release of β-endorphins from the hypothalamus into the cerebrospinal fluid, along with modulating serotonin transmission via serotonergic and norepinephrinergic system mechanisms. Signal transduction from the dermis to the hypothalamus occurs through Aβ and Aδ afferent sensory fibers leading into the spinal dorsal horn (9). As β-endorphins are believed to have antiemetic effects, there is a clear neurobiological basis for APu’s antiemetic effects. Majholm et al. studied the use of acupressure applied at the P6 acupoint, located two cuns (4 cm) proximal to the transverse crease between the tendons of the carpi radialis and palmaris longus. The study found the wristbands to have no antiemetic effect against placebo. Additionally, the study reported mild side effects in a third of the study sample, namely redness, tenderness, and swelling (13). Turgot et al. reported more favorable results in female patients undergoing gynecological procedures. The study found a statistically significant reduction in PONV with the addition of bilaterally applied acupressure wristbands (25). The method of reducing PONV may be more feasible than either APu or EAPu due to the short amount of time necessary to treat each patient, with no special training needed for wristband application.

### Acupuncture

With correct training in traditional Chinese healing techniques, APu at the same P6 acupoint has been shown to be useful in preventing early PONV, postdischarge nausea and vomiting, and alleviating postoperative pain (26, 27). Korinenko et al. presented a randomized, controlled study wherein both the incidence as well as the severity of PON after cardiac surgery was reduced significantly by pre-operative APu. Lee and Done demonstrated APu to prevent PONV as effectively as antiemetic therapy (22). Use of APu was also shown to increase the time to the first postoperative opioid medication use in total hip arthroplasty and abdominal surgery patients (28, 29). As opioids have been linked to opioid-induced nausea and vomiting, the dual antiemetic and analgesic properties of APu seem particularly favorable in the surgical setting, reducing side effects and costs (30). APu does present mild risks. White et al. compiled side effects seen in 32,000 APu consultations within the UK. The authors concluded that mild bleeding and needle pain were the only common complications of APu (26). These findings suggest that only bleeding disorders and extreme anticoagulation therapy should be considered contraindications for prophylactic APu therapy. Further studies are needed to determine whether pre-operative, interoperative, or postoperative APu is most effective at attenuating PONV.

### Electro-Acupuncture

In 1767 at the Middlesex Hospital of London, a special “apparatus” was used to treat patients with electricity. In his book written in 1892, Steavenson mentioned the therapeutic use of electricity at Guy’s Hospital of London around the turn of the century (31). The EAPu practitioner does not have to be as precise when using this method with newer, needleless techniques and a larger area of stimulation (32). New devices such as the ITO ES 130 (ITO Co. LTD., Tokyo, Japan), induce an electrical current in an APu needle, placed at the P6 accupoint. A second needle, placed at a neutral point acts as the grounding electrode, allowing for current to flow through the skin. As with APu, EAPu provides analgesic and antiemetic effects through release of opioid neuropeptides and modulation of the serotonergic, norepinephrinergic, tachykininergic, and endogenous endorphin systems (29, 33). Huang et al. reported a decrease in PONV (*P* = 0.044) corresponding to a transcutaneous electrical acupoint stimulation at 100 Hz, and a lower postoperative visual analgesia score (*P* = 0.047) with frequencies of 2/100 Hz. Reduced visual analgesia scores may in turn be responsible for a decrease in postoperative opioid consumption, known as one of the four major risk factors for PONV (9, 34). High versus low frequency electrical stimulus seems to function differently within the brain. Ji-Sheng Han, noted lesioning of the arcuate nucleus in the hypothalamus eliminated the analgesic effects of low but not high frequency EAPu whereas lesioning of the parabrachial nuclei eliminated high but not low frequency EAPu analgesia. The review also stated that low frequency stimulated opioid neuropeptides mainly bound μ and δ opioid receptors where high frequency stimulated opioid peptides primarily bound κ opioid receptors (29, 33). In clinical trials, Hoffman et al. implemented EAPu therapy for PONV in children between the ages of 4 and 18 years during the postoperative period. The study saw significant reduction in PON (60% in the treatment arms versus 85% in the control arm). POV was not significantly reduced (29). This study demonstrated the feasibility of EAPu in the perioperative setting to reduce PON risk.

In 2014, Yeoh reported that the use of EAPu in laproscopic surgery patients might be a useful, non-pharmacologic intervention for preventing PONV for up to 12 h in adult surgical populations (35).

A study performed at Duke University Medical Center using EAPu stimulation or ondansetron versus placebo for the prevention of PONV concluded that electrostimulation or ondansetron administration encountered the same degree of patient satisfaction and was more effective than placebo (36).

## Conclusion

Alternative therapies offer attractive methods for managing PONV. However, with the exception of APu and EAPu, there is limited evidence supporting the efficacy of complimentary therapy in clinical practice. In order to expand alternative, complementary therapy in the clinical setting, more prospective clinical trials need to be conducted. These non-pharmacological modalities of treatment contribute to an overall patient wellbeing, assisting in physical and emotional healing. Our review of alternative medicine publications conclude that non-medical therapies for PON and POV should be used in addition to medical therapies, rather than as a substitute.

## Author Contributions

NS, NJ, and RH participated in a review of the literature. NS and NJ prepared the body of the manuscript. TG, AU, JP, RH, and SB contributed significantly to the critical review of the text. All authors read and approved the final form of the manuscript.

## Conflict of Interest Statement

The authors declare that the research was conducted in the absence of any commercial or financial relationships that could be construed as a potential conflict of interest.
